# Carbon Nanotubes Enhance the Radiation Resistance of bcc Iron Revealed by Atomistic Study

**DOI:** 10.3390/ma12020217

**Published:** 2019-01-10

**Authors:** Shuzhuang Liu, Lu Xie, Qing Peng, Rui Li

**Affiliations:** 1 School of Mechanical Engineering, University of Science and Technology Beijing, Beijing 100083, China; shuzhuang1993@163.com (S.L.); xielu@ustb.edu.cn (L.X.); 2 Nuclear Engineering and Radiological Sciences, University of Michigan, Ann Arbor, MI 48109, USA

**Keywords:** irradiation, carbon nanotube/Fe composite, He bubbles, point defects

## Abstract

With extra space, a carbon nanotube (CNT) could serve as an absorber of point defects, including helium interstitials, and outgas the accumulate helium via “nano-chimneys”. The radiation resistance of CNT/Fe has still not been fully understood. Herein, we investigated the influence of CNTs on low-energy helium irradiation resistance in CNT/Fe composites by molecular dynamic simulations. CNTs reduced the small and medium He clusters in the Fe matrix. When the incident energy of the He atoms was 300 eV, the He atoms aggregated at the outer surface of CNTs. CNTs postponed the formation of He bubbles. When the irradiation energy was higher than 600 eV, He atoms could penetrate the walls of CNTs and form clusters inside the single-walled CNTs or the space in double-walled CNTs—the latter presented better performance. The reduction of Frenkel pair point defects suggested the enhancement of radiation resistance by the presentation of CNTs. Our results might be useful for the material design of advanced steels for radiation resistance.

## 1. Introduction

The accumulation of helium bubbles is one of the main threats to the safety in operating reactors. As a main product, He atoms may form bubbles in materials, which result in swelling, voids, cracks, and embrittlement [1,2] of the structure materials. He irradiation resistance performance of structural materials is crucial in first wall material design in nuclear fusion reactors. Steels are undoubtedly important structural materials in nuclear engineering and fusion engineering [3]. There is a continuing demand to improve the irradiation resistance of steel. Previous efforts suggested that interfaces and grain boundaries in composites are beneficial to the radiation resistance of metals [4,5]. For instance, the disperse oxides in oxide dispersion strengthened (ODS) steel formed a large number of dislocation grain boundaries, which improved its radiation resistance [6]. In addition, both nanocrystalline materials [7] and nanofoams [8] were proven to have good radiation resistance.

Carbon nanotubes (CNTs) [9] have attracted extensive interest from researchers because of their excellent electrical, mechanical, and thermal properties [10,11,12]. They have been widely used as reinforcements in composite materials due to their excellent mechanical properties [13,14,15]. It has been proven that carbon nanotubes enhanced the strength, hardness, and wear properties of metals [16,17,18]. Recently, researchers found that carbon nanotubes might also improve the irradiation resistance performance of matrix structures. Kang et al. [19] indicated that 0.5 wt % CNTs in CNT/Al composites improved the tensile strength without the reduction of tensile ductility before irradiation in addition to reducing void/pore generation and radiation embrittlement at high displacements per atom (DPA). They speculated that one dimensional surviving carbon nanostructures might also form percolating paths of “nano-chimneys” that evict the accumulated He and other fission gases. It was reported that CNTs improved the radiation resistance of CNT/Ni composites by a mechanism of He trapping around CNTs [20]. Besides CNTs, graphene has also attracted researchers’ attention [21,22]. An atomistic study on the irradiation damage and interface stability of copper/graphene nanocomposites showed that the point defects in nanocomposites were reduced under low primary knock-on atom (PKA) energy—however, the radiation damage resistance of the composite might be weakened if higher PKA energy occurred [23]. Ultrahigh-strength V-graphene nanolayers were demonstrated to have an excellent radiation tolerance to He ion irradiation [24]. This was because the graphene interface absorbed radiation defects spontaneously caused by cascade collisions before achieving self-repair. A He ion implantation experiment in graphene/W multi-layer materials revealed that the insertion of graphene both reduced radiation damage and made He bubbles less likely to form [25].

The aforementioned works well-demonstrate that carbon nanomaterials have the potential to enhance the radiation resistance of metals. However, the direct kinetics of He irradiation on carbon nanotube/metal composites have not been reported to the best of the authors’ knowledge. The dynamics of He accumulation and defect evolution in the composite under accumulated irradiation is not clear—especially under low energy He irradiation, which might also lead to He bubble formation [26]. Here, we investigated the low-energy He irradiation damage to bcc-Fe [27] and CNT/Fe composite by classic molecular dynamics simulations. Both the migration and aggregation of He in the Fe and CNT/Fe composite and the evolution of point defects in the matrix were studied. The influence of carbon nanotubes on the irradiation damage resistance was evaluated.

## 2. Materials and Methods

**Figure 1:**
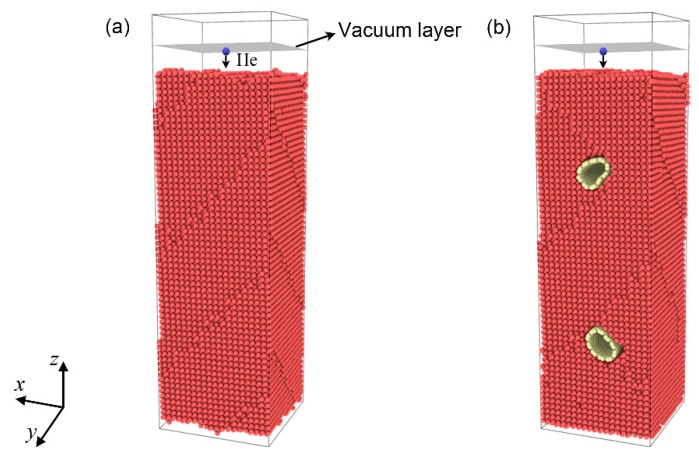
Simulation models: (**a**) bcc-Fe; (**b**) single-walled carbon nanotube (SWCNT)/Fe composite. The red, yellow, and blue balls denote the Fe, C, and He atoms, respectively.

Simulation models for pure bcc-Fe and a single-walled CNT (SWCNT)/Fe composite were used, as shown as Figure 1a,b, respectively. The dimensions of bcc-Fe (0 0 1) were 6.02 nm, 6.02 nm, and 17.48 nm along the *x*, *y*, and *z* directions, respectively. The number of Fe atoms was 50,901. We modeled the SWCNT/Fe composite with two SWCNTs (10, 10) embedded in the bcc-Fe (0 0 1) matrix (Figure 1b). The diameter of SWCNTs (10, 10) was 1.37 nm. The axes of SWCNTs (10, 10) were parallel to the *y* direction and spanned the entire matrix. The two SWCNTs were arranged in a symmetrical pattern. The centroids of the upper and lower SWCNTs in the *z* direction were about 5.0 nm away from the upper and lower surface of the Fe matrix, respectively. The dimensions of the composite in the *x*, *y*, and *z* directions were the same as those of the Fe matrix in Figure 1a. The number of Fe atoms and C atoms was 49,096 and 1916, respectively. To avoid movement of the substrate owing to momentum from the incident He atoms, the three bottom-layer Fe atoms in the Fe substrate were fixed. Periodic boundary conditions were applied along both the *x* and *y* directions in the two models. To conduct the same periodic boundary for the carbon nanotube and Fe substrate in the *y* direction in model b, the Fe substrate was compressed 0.13% along the *y* direction. The influence of this compression was verified to be trivial.

A mixed Tersoff-ZBL potential was applied to describe the interactions among Fe and C atoms and was successfully used to study the interaction of dislocations with carbides in bcc-Fe [28,29]. The interactions between He, Fe, and C atoms were used by the ZBL [30] potential. The Beck potential [31] was employed to describe the interaction between He atoms [32]. All the molecular dynamic simulations were performed by LAMMPS. To embed SWCNTs (10, 10) in the Fe matrix, 1805 Fe atoms were removed to make room for the SWCNTs. The SWCNT/Fe composite structure was obtained from the annealing process with the NPT ensemble. The time step was 1 fs. The initial SWCNT/Fe structure was relaxed at 300 K for 100 ps before heating up to 2000 K at a rate of 34 K/ps. Then, the system was maintained at 2000 K for 1000 ps. Finally, the system temperature went down to 300 K at a rate of 34 K/ps and was maintained for 100 ps. The final structure of the SWCNT/Fe composite after the annealing process showed that the SWCNTs (10, 10) kept their hollow structures. The Fe matrix was also conducted with the same annealing process. After obtaining the initial structure, the simulation systems were relaxed to reach the equilibrium. An NVT ensemble with a temperature of 300 K—controlled by a temperature rescaling thermostat—was applied. The time step was 1 fs. The relaxation process was held for 100 ps. The structure was then ready for further irradiation damage study. The ensemble for irradiation damage was still an NVT with a temperature of 300 K. The thermostat was changed to Berendsen. He atoms were randomly generated in a 0.2-nm-thick vacuum layer that was 1 nm away from the top surface of the substrate and then bombarded toward the matrix. Three incident energies of the He atoms—300 eV, 600 eV, and 900 eV—were applied because we focused on the formation of characteristic damage features under low-energy irradiation, which is still not clear. In order to further analyze the trajectories of the He atoms and the defects in the matrix, the visualization software VMD [33] and OVITO [34] were used.

## 3. Results and Discussions

### 3.1. The Migration and Aggregation of He in the Matrix

**Figure 2:**
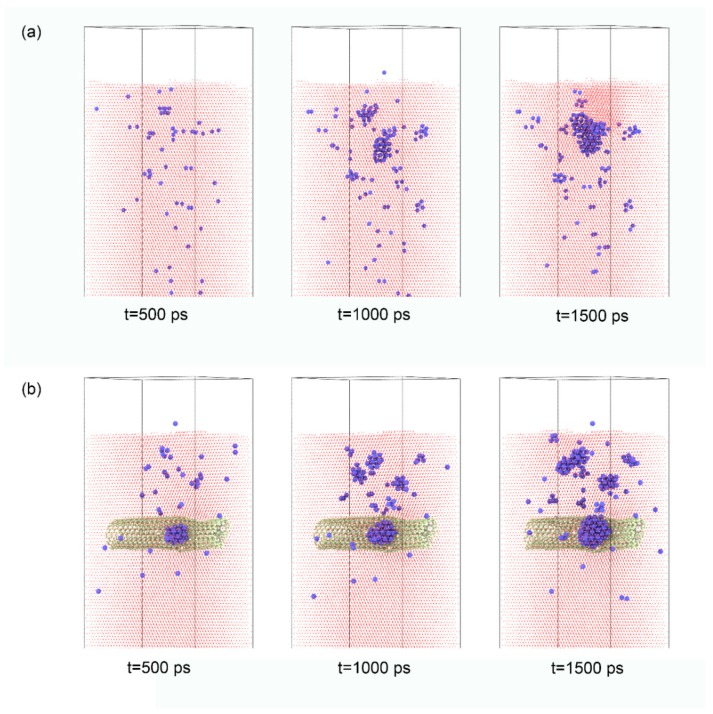
He clusters in the matrix when the incident energy of the He atoms was 300 eV. (**a**) Fe matrix; (**b**) SWCNT/Fe composite. The blue and yellow balls denote He and C atoms, respectively.

**Figure 3:**
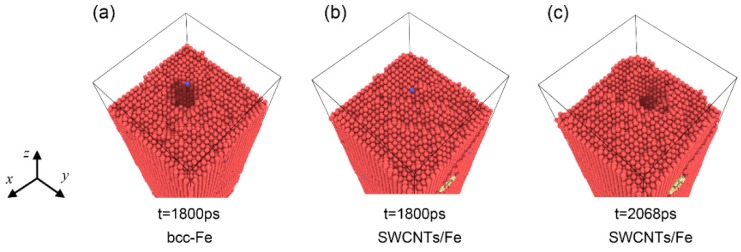
Surface topography of the matrix under irradiation. (**a**) He bubble burst from the Fe matrix, t = 1800 ps; (**b**) the surface topography of SWCNT/Fe composite, t = 1800 ps; (**c**) He bubble burst from the SWCNT/Fe composite, t = 2068 ps.

As with the first step, several tests were conducted to optimize the depth of the SWCNTs. If the location of the upper SWCNT was deeper than 6.5 nm, He would not interact with the SWCNT when the incident energy was 300 eV. At last, the depth of the upper SWCNTs was set 5.0 nm away from the upper surface of the Fe matrix. The snapshots of He atoms in the Fe matrix and the SWCNT/Fe composite are displayed in Figure 2. The corresponding times were 500 ps, 1000 ps, and 1500 ps when the incident energy of the He atoms was 300 eV. For the Fe matrix in Figure 2a, the He atoms initially scattered in the upper part of the matrix (t = 500 ps) before aggregating to form big clusters (t = 1000 ps), which then formed the He bubble (t = 1500 ps). After that, the He bubble continued to grow by capturing the new incident He atoms. When the irradiation time reached 1800 ps, the He bubble burst from the Fe matrix and left a hole in it, which resulted in irreversible damage in the matrix, as shown in Figure 3a. For the SWCNT/Fe composite, the migration and aggregation of He atoms were different. As shown in Figure 2b, He atoms gathered quickly at the interface between the SWCNTs and Fe substrate, and a small He cluster formed there (t = 500 ps). At t = 1000 ps—though several small clusters appeared in the matrix—the largest cluster was on the CNT surface. When t = 1500 ps, a He bubble formed at the interface. The results show that the interface between carbon nanotubes and the Fe matrix could trap He atoms. A similar result was obtained in a previous study of a CNT/Ni composite [20]. Referring to the bcc-Fe, the He clusters in the SWCNT/Fe composite were scattered and smaller at the irradiation time of 1500 ps. When the He bubble in the Fe matrix burst at t = 1800 ps, the structure of the SWCNT/Fe composite remained stable, as shown in Figure 3b. The bubble at the interface between the carbon nanotube and Fe substrate burst finally at t = 2068 ps, as shown in Figure 3c, which implied that the SWCNT/Fe composite could delay the formation of bubbles and therefore enhance the He irradiation tolerance.

**Figure 4:**
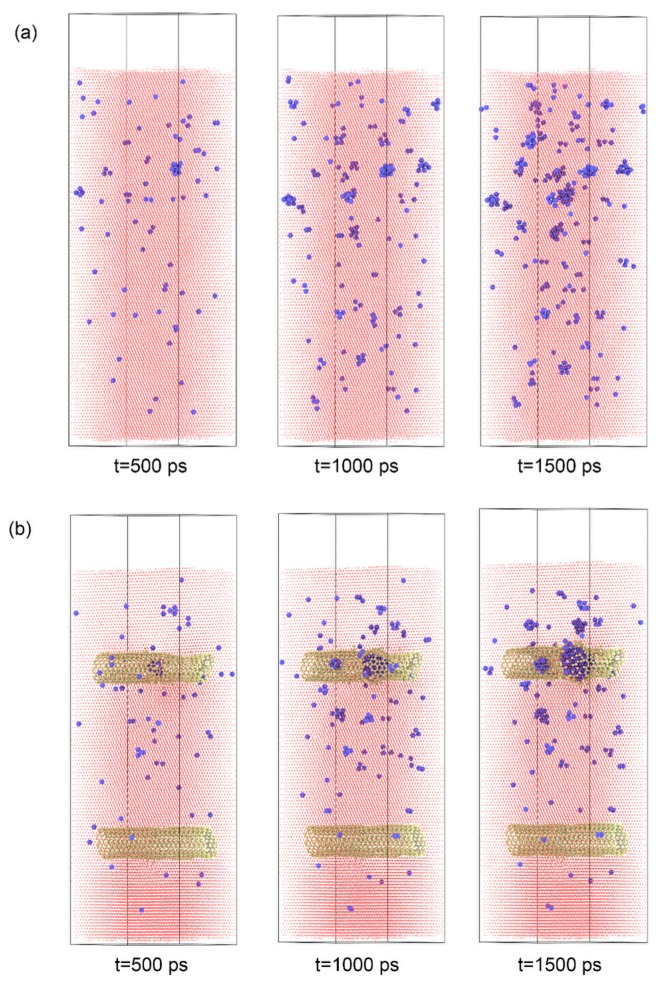
He clusters in the matrix when the incident energy of the He atoms was 600 eV. (**a**) Fe matrix; (**b**) SWCNT/Fe composite. The blue and yellow balls denote He and C atoms, respectively.

When the incident energy of the He atoms was 600 eV or 900 eV, the He atoms penetrated the upper carbon nanotube and aggregated inside it. In these two cases, the migration and aggregation behaviors of He atoms were similar. Therefore, we only discussed the case in which the incident energy of the He atoms was 600 eV in detail. The snapshots of He atoms in bcc-Fe and the SWCNT/Fe composite are shown in Figure 4a,b, respectively. He atoms would go deeper in the matrix under higher irradiation, and they would scatter in the matrix (Supplementary movie S1) when the incident energy of the He atoms was 600 eV, instead of forming large clusters. Only small and medium clusters formed in the Fe matrix at t = 1500 ps. After penetration, He atoms began to aggregate inside SWCNTs, as shown in Figure 4b. At t = 1000 ps, He clusters were created. A He bubble formed inside the carbon nanotube at t = 1500 ps, and the structure of the SWCNT was damaged. The SWCNTs served as a He sink that absorbed the incident He atoms, resulting in the reduction of He atoms in the matrix (Supplementary movie S2).

**Figure 5:**
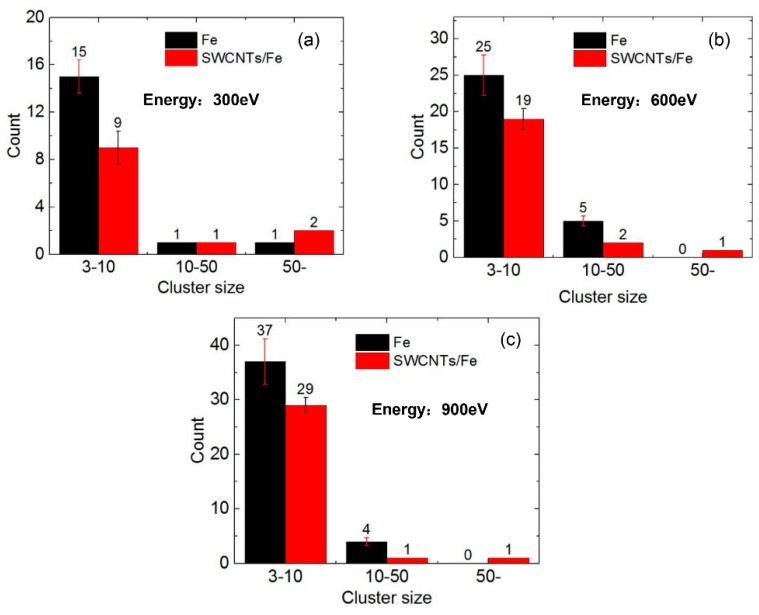
The number of He clusters in the Fe matrix and the SWCNT/Fe composite, t = 1200 ps. The incident energies of the He atoms were (**a**) 300 eV; (**b**) 600 eV; and (**c**) 900 eV.

The number of He clusters in the Fe matrix and the SWCNT/Fe composite at t = 1200 ps are shown in Figure 5a–c when the incident energy of the He atoms was 300 eV, 600 eV, and 900 eV, respectively. The clusters were divided into three categories: small clusters (3–10 He atoms), medium clusters (10–50 He atoms), and He bubbles (more than 50 He atoms). There were 15 small clusters, one medium cluster, and one bubble on average in bcc-Fe (Figure 5a) if the incident energy was 300 eV. When the incident energies were 600 eV and 900 eV, there were 25 and 37 small clusters and five and four medium clusters, respectively. There were no He bubbles observed. For the SWCNT/Fe composite, small and medium clusters decreased obviously under all three incident energies compared to those in bcc-Fe. He bubbles at the interface between a SWCNT and the Fe matrix rose to two when the incident energy was 300 eV. One bubble was observed inside the upper SWCNT in two cases, with the incident energies at 600 eV and 900 eV. Therefore, it could be inferred that the SWCNTs had a strong effect on trapping He atoms.

### 3.2. Point Defects in Fe Matrix and SWCNT/Fe Composite

**Figure 6:**
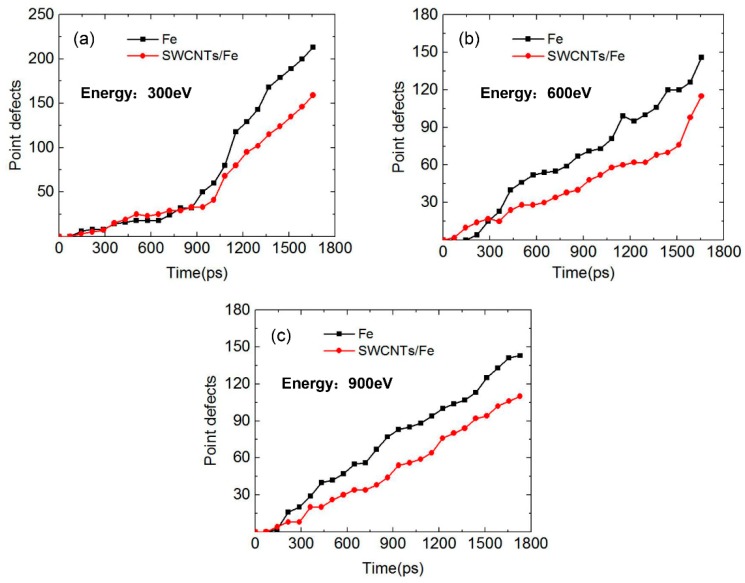
The number of point defects in Fe and SWCNT/Fe during the irradiation process. The incident energies of the He atoms were (**a**) 300 eV; (**b**) 600 eV; and (**c**) 900 eV.

The Wigner-Seitz [35] (WS) defect analysis method was applied to calculate the point defects. The relaxed structures of the Fe matrix and the SWCNT/Fe composite were taken as the reference structures for WS analysis. Each WS cell in the reference structure contains exactly one atom. If a WS cell in the target system is empty, it is a vacancy. If a WS cell contains more than one atom, it is an interstitial-type defect. The number of point defects (vacancies and interstitials) in Fe and SWCNT/Fe under three incident energies are shown in Figure 6. Basically, the number of point defects increased as He atoms bombarded the matrix continuously. When the incident energy of the He atoms was 300 eV (Figure 6a) during the first 900 ps, the defects in the two structures were less and the numbers were almost the same. After 900 ps, the number of defects increased significantly, which indicates that He clusters were generated, resulting in a large displacement of the substrate atoms—all of which results in a large number of defects. The number of defects in the SWCNT/Fe composite was less than that in bcc-Fe. When the incident energy was 600 eV, the number of defects in the composite was larger than that in bcc-Fe in the first 300 ps. After 300 ps, it showed the opposite trend. When it came to 900 eV, the transition point of the trend was about 150 ps. In general, the number of point defects in the SWCNT/Fe composite was larger than that in bcc-Fe during long-term bombardment. This was because the interface between the carbon nanotubes, the substrate, and the internal space of the carbon nanotubes could trap He atoms. The collection of atoms reduced the cascade collision and the generation of point defects.

**Figure 7:**
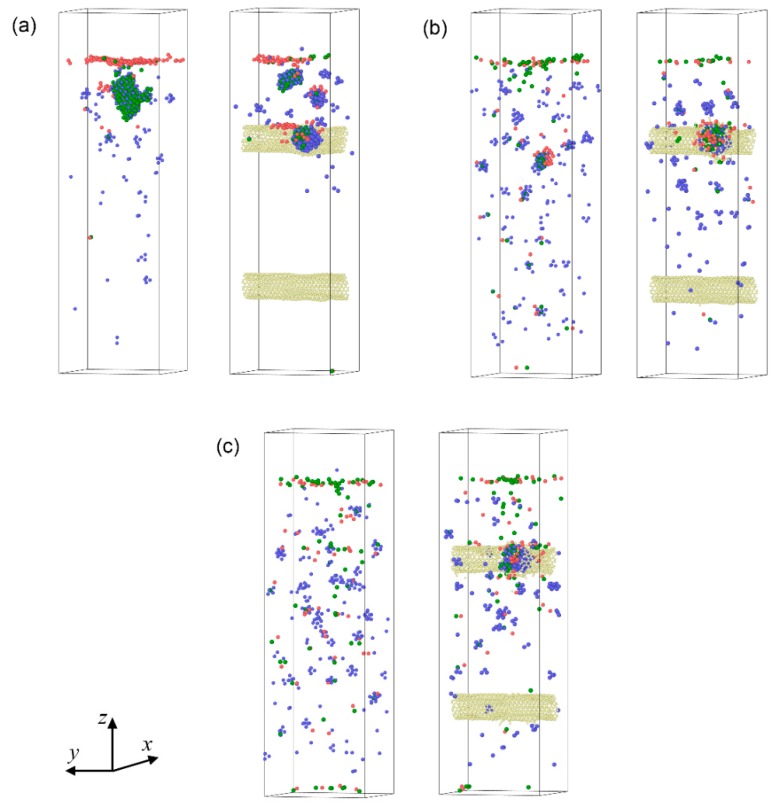
Point defects distribution in the Fe matrix and the SWCNT/Fe composite, t = 1200 ps. The left and right sides of (**a**), (**b**), and (**c**) represent the Fe matrix and the SWCNT/Fe composite, respectively. The red and green balls denote interstitials and vacancies, respectively. The blue and yellow balls denote He atoms and C atoms on the carbon nanotubes, respectively. The incident energies of the He atoms were (**a**) 300 eV; (**b**) 600 eV; and (**c**) 900 eV.

The spatial distribution of point defects in bcc-Fe and the SWCNT/Fe composite at t = 1200 ps are shown in Figure 7a–c for incident energies of 300 eV, 600 eV, and 900 eV, respectively. When the incident energy was 600 eV, He bubbles formed at the upper part of the Fe matrix, which resulted in a cluster of vacancies in the matrix (as shown by the green balls in Figure 7a). At the same time, Fe atoms moved toward the surface and were out of initial sites, which led to plenty of interstitials in positions near the surface. If the incident energy was higher, as shown in Figure 7b,c, point defects tended to occur in deeper sites. The defects near the upper SWCNTs were notable. It could be inferred that SWCNTs played an important role in trapping point defects.

### 3.3. The Effect of Double-Walled Carbon Nanotube (DWCNT)/Fe Composite

**Figure 8:**
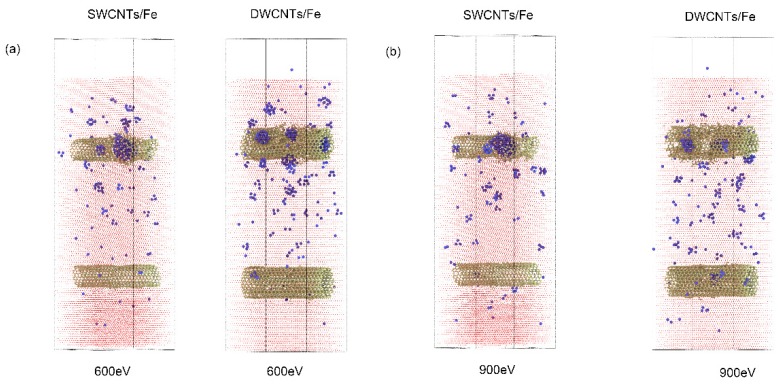
The distribution of He clusters in SWCNT/Fe and double-walled carbon nanotube (DWCNT)/Fe composites. The incident energies of the He atoms were (**a**) 600 eV; and (**b**) 900 eV.

Considering that multi-walled carbon nanotubes (MWCNT) have broad applications, the effect of double-walled carbon nanotubes (DWCNTs) on He irradiation resistance was also evaluated. The inner and outer tubes of DWCNTs were CNTs (10, 10) and CNTs (15, 15). The corresponding diameters of the tubes were 1.37 nm and 2.06 nm. The depth of the DWCNTs was the same as the model with SWCNTs, and the other parameters in the model were also consistent with the previous model. When the incident energy of the He atoms was 300 eV, He clusters tended to form between the outer surface of the DWCNTs and the Fe matrix, which was similar to the model with SWCNTs. However, when the incident energies of the He atoms were 600 and 900 eV, different phenomena appeared between the model with DWCNTs and the model with SWCNTs. Figure 8 shows the distribution of He clusters in the Fe matrix in those two kinds of models when the incident energies were 600 eV and 900 eV. If SWCNTs were presented, He bubbles formed inside the tube and the structure of the SWCNTs was damaged. For the model with DWCNTs, smaller He clusters were scattered at the outer, inner, and between such tubes of the DWCNTs. This phenomenon could be explained as follows: the space between the outer and inner tubes of CNTs offered more spots for He to form clusters, which decreased the possibility to form big He clusters.

**Figure 9:**
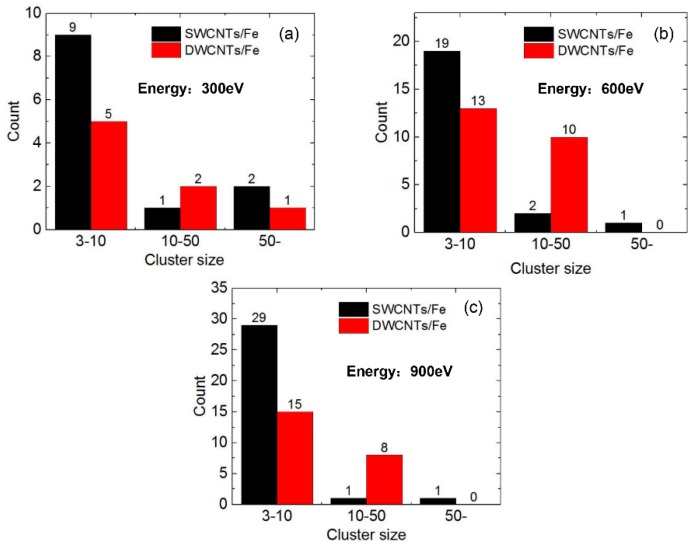
The number of He clusters in SWCNT/Fe and DWCNT/Fe composites, t = 1400 ps. The incident energies of the He atoms were (**a**) 300 eV; (**b**) 600 eV; and (**c**) 900 eV.

Figure 9 shows the comparison of the numbers of He clusters between the model with SWCNTs and that with MWCNTs at the later stage of simulation—t = 1400 ps—when the incident energy of He was 300, 600, and 900 eV, respectively. The results showed that there were no He clusters whose size were bigger than 50 in the model embedded in DWCNTs when the incident energies of He were 600 and 900 eV. The number of small clusters with number sizes between 3 and 10 decreased, and the number of medium clusters with sizes between 10 and 50 increased, which indicated that He atoms tended to form medium clusters—not big He bubbles—in the outer and inner tubes of DWCNTs, which could further postpone the bursting of He bubbles. A similar conclusion could be expected when MWCNTs are applied. Thus, a MWCNT/Fe composite would have better effect on irradiation resistance.

## 4. Conclusions

The kinetics of He irradiation on both bcc-Fe and CNT/Fe composites under low incident energy was studied by molecular dynamics simulations. The results showed that the small (3 ≤ n < 10) and medium (10 ≤ n ≤ 50) He clusters in the SWCNT/Fe composite were less than those in bcc-Fe. When the incident energy of the He atoms was relatively low (300 eV), He atoms tended to aggregate at the interface between SWCNTs and Fe. The SWCNTs retarded the burst of He bubbles, which implied the enhancement of the irradiation resistance of the SWCNT/Fe composite under low irradiation energy. If the incident energy of the He atoms was higher than 600 eV, He atoms penetrated the SWCNTs and aggregated inside the SWCNTs. SWCNTs thus served as a sink to trap He atoms, leading to the radiation swelling resistance of the SWCNT/Fe composite. In addition, the point defects of Fe in the SWCNT/Fe composite were much less than those in bcc-Fe, demonstrating the enhancement of the radiation resistance. The point defects were notable near SWCNTs, indicating that the SWCNTs also served as sinks to Fe vacancies and interstitials. The results reveal that CNTs played a significant role in trapping defects and decreased irradiation damage. When DWCNTs were embedded in the Fe matrix, the space between the outer and inner tubes offered more spots to form He clusters. More medium He clusters formed, and big He clusters decreased obviously compared to the model with SWCNTs, which indicated the better performance of irradiation resistance of the DWCNT/Fe composite. This study suggests that the CNT/Fe composite could be an advanced radiation-resistant structural material, in addition to having outstanding mechanical properties.

## Supplementary Materials

The following are available online at http://www.mdpi.com/1996-1944/12/2/217/s1, Video S1: Movie S1.avi. Video S2: Movie S2.avi.
